# Effects of left-hand contraction on tennis serve performance

**DOI:** 10.3389/fpsyg.2024.1386025

**Published:** 2024-05-07

**Authors:** Kanta Mizuno, Hiroaki Masaki

**Affiliations:** ^1^Graduate School of Sport Sciences, Waseda University, Saitama, Japan; ^2^Faculty of Sport Sciences, Waseda University, Saitama, Japan

**Keywords:** left-hand contraction, choking under pressure, clutch, tennis serve, wearable grasping material

## Abstract

**Introduction:**

The tennis serve is commonly executed in high-pressure scenarios, often leading to performance decline; a condition commonly referred to as choking under pressure. One suggested effective method to avert choking involves contracting the left hand. We examined the effects of left-hand contraction on tennis serve performance using a wearable grasping material (polyurethane foam) which can be incorporated into sportswear.

**Materials and methods:**

We assigned 40 right-handed skilled tennis players to either the contraction group (*n* = 20) or the no-contraction group (*n* = 20). They were instructed to perform a second-serve task during the pre-test and pressure test. The participants in the contraction group squeezed the grasping material for 20 s before executing the task in the pressure test. We measured performance, including total scores, the number of maximum score achievements, landing positions, and kinematic indices (i.e., ball speed, racket speed, and impact height).

**Results:**

Although neither group demonstrated deteriorated performance on the pressure test, the contraction group experienced an increased number of maximum score achievements under the pressure situation compared with the pre-test (*p* = 0.021).

**Discussion:**

Our results suggest that when under pressure, left-hand contraction may improve performance during tennis serves.

## 1 Introduction

Serving stands out as one of the paramount skills in tennis, exerting a direct impact on match results (Whiteside et al., 2013; Kovalchik and Reid, 2017). Notably, the second-serve takes center stage in tense moments, serving as a pivotal strategy to prevent double faults (Weinberg, 2005). Athletes are required to exhibit superior performance in competitive situations. However, psychological pressure—due to a competitive situation, the presence of an audience, reward/punishment contingency, and ego relevance—often deteriorates performance (Baumeister and Showers, 1986). One phenomenon associated with diminished performance is “choking under pressure” (Baumeister, 1984). Although several relaxation techniques (including autogenic training, progressive muscle relaxation, and biofeedback) have been proposed as potential solutions to choking, the acquisition of these techniques requires long-term regular practice and debriefing (e.g., Stetter and Kupper, 2002). Hence, it is vital to develop more effective and easier methods to prevent choking.

By recording electroencephalograms (EEGs), prior studies have demonstrated that experts in closed-skill sports (e.g., golf, rifle shooting, and archery) tend to inhibit neuronal activity in the left hemisphere (indexed as increased alpha power) while stabilizing activation in the right hemisphere prior to performing critical movements (Hatfield et al., 1984; Salazar et al., 1990; Crews and Landers, 1993). The left hemisphere is primarily associated with verbal analysis, whereas the right hemisphere is associated with visuospatial processing (De Renzi, 1982). Notably, verbal-analytical engagement during motor preparation is thought to be indicative of conscious processing of movement (Zhu et al., 2011). Therefore, the left hemispheric inhibition during motor preparation may suggest pre-attentive (i.e., automated) motor control, which is typical of expert performance (e.g., Haufler et al., 2000); meanwhile, the left hemispheric activation disrupts smooth movements (Zhu et al., 2011; Gallicchio et al., 2016), resulting in choking. Given that unilateral muscle contraction of the left hand can activate the right hemisphere while relatively deactivating the activity of the left hemisphere (Harmon-Jones, 2006; Hirao and Masaki, 2019), the left-hand contraction preceding a crucial movement may help to prevent choking.

Indeed, Beckmann et al. (2013) reported that repetitive left-hand contraction could prevent choking in a variety of sports-related skills, including the penalty kick of indoor soccer, the taekwondo kicks, and badminton serves. They found that the left-hand contractions immediately before a critical movement effectively prevented choking under pressure. Conversely, contractions of the right hand prior to tasks have been linked to choking (Beckmann et al., 2013). Subsequent studies have replicated these findings. Gröpel and Beckmann (2017, Study 1) successfully prevented choking during gymnastics matches (i.e., German university championships) by employing left-hand contractions. Additionally, Beckmann et al. (2021) discovered that grasping a tennis ball with the left hand maintained serve performance in competitive situations involving highly skilled tennis players.

Moreover, left-hand contractions have been shown to enhance athletic performance under pressure in various sports such as taekwondo kicking (Beckmann et al., 2013, Study 2), gymnastics (Gröpel and Beckmann, 2017, Study 2), and bowling (Mesagno et al., 2019, Study 2). These instances of improved performance under pressure are commonly referred to as “clutch” phenomena (Otten, 2009), characterized by enhanced performance when athletes perceive pressure-induced anxiety as a challenge (i.e., positive perception) rather than a threat (i.e., negative perception) (Blascovich et al., 1999; Cheng et al., 2009). These findings strengthen the effectiveness of the left-hand contraction for achieving high performance under pressure.

Psychophysiological research has provided valuable insights into how the left-hand contraction influences brain states during the preparation of movements. Deeny et al. (2003) found that highly skilled marksmen exhibited weak EEG connectivity between the left temporal region (T7) and the frontal region (Fz) during the aiming phase. Weak connectivity (i.e., desynchronization) of EEGs may indicate a reduction in the cortico-cortical communications between the left hemisphere (which is responsible for verbal-analytical processing) and the frontal region, which is involved in motor planning (Deeny et al., 2003; Gallicchio et al., 2016). These results indicate that verbal-cognitive (i.e., conscious) motor processing should be inhibited to achieve automated movement execution.

Lo et al. (2019) observed strong T7-Fz connectivity along with performance deterioration in dart throwing, suggesting an elevation in conscious processing under pressure. Hoskens et al. (2020) revealed a reduction in T7-Fz connectivity after the left-hand contraction in a golf putting task, implying that the left-hand contraction may prevent choking by suppressing verbal analysis in the left hemisphere, which involves the conscious processing of movement.

Although the abovementioned studies by Beckmann et al. used a soft ball for the left-hand contraction, they ambiguously described the ball material and grasping manners, making it difficult to conduct follow-up studies. Furthermore, it is difficult to practice ball grasping in competitive situations without interference from subsequent movements. To address this issue, Masaki (2022, mandatory report of a research grant) attempted to incorporate polyethylene foam into sportswear to accomplish left-handed grasping before performing a critical action, even in a competitive situation. Although he did not observe any obvious effect of left-handed grasping on performance scores in the first level of analysis, the kinematic data remained unanalyzed. Given that subtle decreases in performance due to pressure induction tend to manifest only in kinematic indices (Tanaka and Sekiya, 2010), kinematics should be examined in studies on choking prevention. To clarify whether wearable grasping materials can have a beneficial effect on real-game performance, we reanalyzed the kinematic data of Masaki (2022), expanding earlier results and adding new findings.

Compared to beginner-level tennis serves, those of experts are characterized by faster ball speeds, higher impact heights, and earlier peak activities of electromyograms (EMGs) in the leg extensor muscles during the serving phase (Girard et al., 2005). Intermuscular coordination that enables adequate energy transfer may indicate the efficiency of a tennis serve (Girard et al., 2005). Additionally, high spatial resolution analysis has provided precise distribution of ball landing positions, confirming that elite tennis players can serve a ball closer to a target position than recreational players (Hernández-Davó et al., 2019).

Psychological pressure may impair coordinated movements. Both decreased amplitude and speed of action have been observed under pressure conditions (e.g., golf putting, Tanaka and Sekiya, 2010; table tennis, Sekiya and Tanaka, 2019). These findings suggest that kinematic variables are vulnerable to psychological pressure. Therefore, it is important to measure kinematics to thoroughly evaluate motor impairment due to pressure (Sekiya and Tanaka, 2019). Additionally, kinematic change may result in high variability in landing positions. The heightened variability in error distribution under pressure (Ellis and Ward, 2022) can be assessed using two-dimensional coordinate analysis, a method that examines performance consistency (Hancock et al., 1995).

The aim of this study was to investigate the impact of left-hand contraction on tennis serve performance, utilizing a practical method involving grasping a material for sportswear. Kinematic variables such as ball and racket speed and impact height were measured. Our hypothesis posited that left-hand contraction would maintain serve performance, including scores and landing positions, even under pressure, despite an increase in state anxiety and potential deterioration in serve qualities. We also anticipated that the left-hand contraction would conserve appropriate kinematics (i.e., the high speed of served balls and racket movements, and high impact heights) in a pressure situation. Conversely, we expected performance deterioration under pressure in the control condition (i.e., no left-hand contraction), which would disrupt these kinematic variables.

## 2 Materials and methods

We report a secondary analysis of the preliminary analysis conducted by the last author. A portion of the results were published in an extremely limited and mandatory report written in Japanese to complete a research grant (Masaki, 2022). In the preliminary analysis, we only analyzed performance scores and subjective anxiety ratings. In this study, we explored the number of successes, landing positions, and kinematics. The outcomes of the secondary analysis are worth reporting and are statistically significant.

### 2.1 Participants

We recruited 40 skilled tennis players (*M* = 20.0 yrs, *SD* = 1.2) from a university tennis team. All participants had more than 3 years of tennis experience, with the majority having competed in intercollegiate tournaments. Participants were randomly assigned to either the contraction group (11 males and nine females) or the no-contraction group (12 males and eight females). Each group comprised 17 intercollegiate-level and 3 regional-level players. They were right-handed, as assessed by the Edinburgh Handedness Inventory (*M* = +92.7, *SD* = 16.9; Oldfield, 1971). With a sample size powered at 0.80 to detect significance at an alpha level of 0.05, G*Power (Faul et al., 2007) determined a required sample size of 34 for a repeated measures analysis of variance (*f* = 0.25), corresponding to a small to medium effect size (Cohen, 1992). This study was approved from the local ethics committee (approval number: 2020–077).

### 2.2 Experimental task

**Figure 1:**
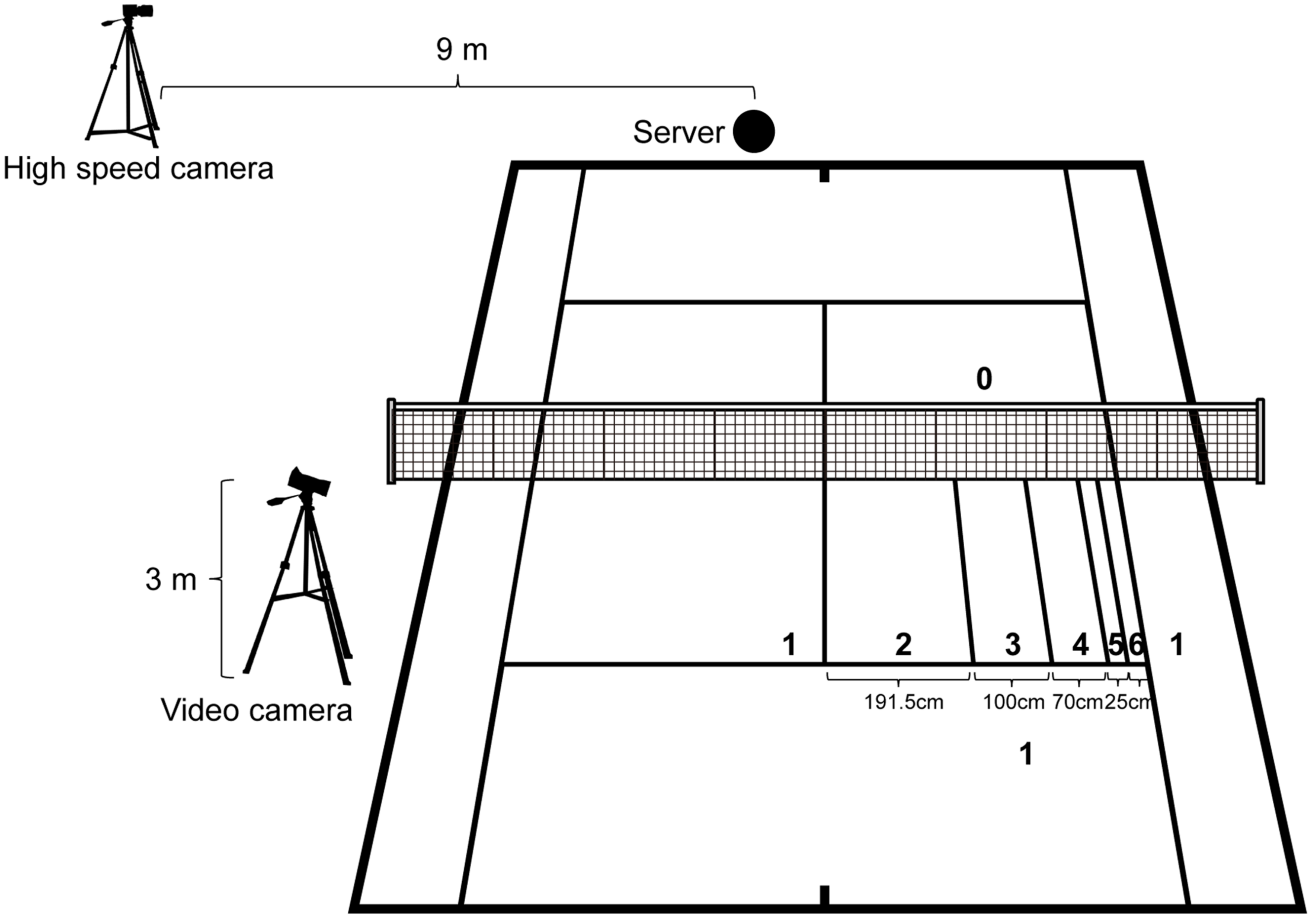
The experimental setup. The numbers denote the point in question.

The participants were instructed to serve a ball from a predetermined position (i.e., 1 m to the right of the center mark), targeting the wide corner of the deuce service court. We quantified serving scores based on the ball landing position, as classified by the lines drawn on the court (Figure 1). We established scoring zones across the entire service court. Following assessment by a skilled tennis player, we adjusted the scoring zones to align with difficulty levels, as proficient serves typically target the near side-line in the wide corner. The participants were told to “perform second serves as if they were in a real match, aiming for maximum accuracy to achieve the highest possible score.” We presumed that the second serve would be executed under pressure because its failure would result in losing a point in real tennis competitions as a double fault (Weinberg, 2005). The tasks were performed using their own rackets. Faults received a score of 0 points. If a let occurred, an additional serve was provided, adhering to official tennis rules. Serving motions were recorded using a high-speed camera positioned 9 m away that captured the right sagittal plane. The courts were recorded at a height of 3 m.

### 2.3 Procedure

**Figure 2:**
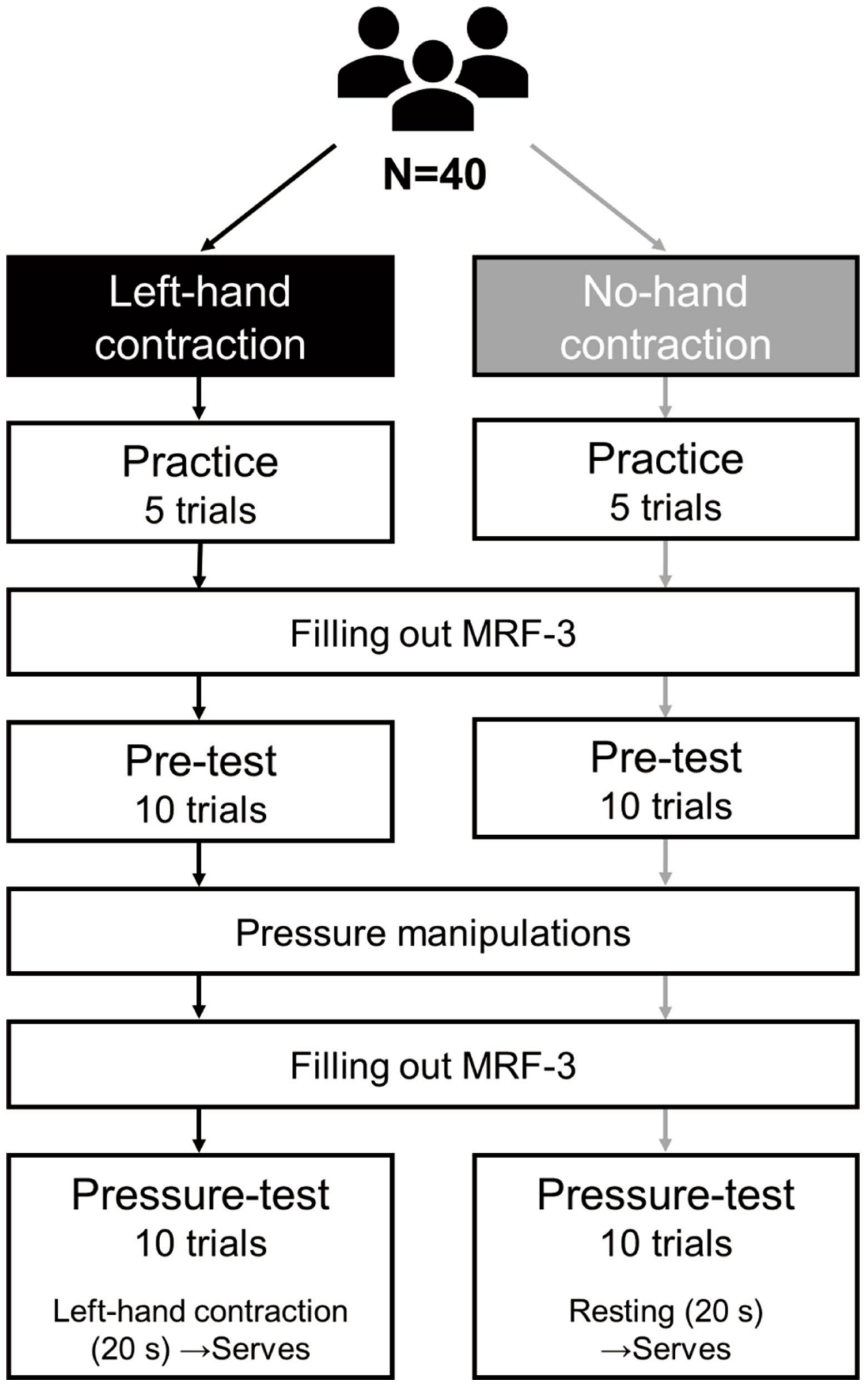
Study protocol.

The participants attended a 30-min session. After receiving instructions for the experiment, they completed the serving task, which comprised 25 servers. This consisted of 5 serves in the practice session, 10 on the pre-test, and 10 on the pressure test. Before each test, we assessed state anxiety (Figure 2).

**Figure 3:**
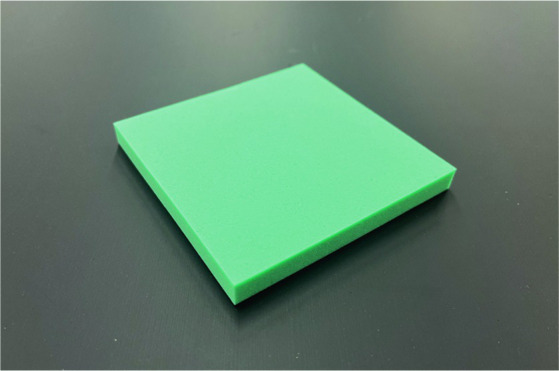
Grasping material (expanded polyethylene foam).

On the pre-test, participants in both groups served balls without hand contractions. On the pressure test, participants in the contraction group served a ball immediately after contracting their left hand by grasping a material called “expanded polyethylene foam” (0.1 m × 0.1 m × 0.01 m, density 30 kg/m^3^, compressive stress 50 kPa, P0030, manufactured by Fuji Gomu Co., Japan, Figure 3). This material was embedded in a flat pocket that could be sewn into the sportswear. They repeatedly grasped the material with their left hand for 20 s immediately before serving. Participants in the no-contraction group did not grasp the material during the same period (20 s). We set the grasping pace at 60 beats per minute (bpm) and recorded the sounds using a metronome.

Before the pressure test, the participants were told the following, which was effective for inducing state anxiety as in previous studies (Cooke et al., 2014; Lo et al., 2019): (a) the serving motion would be recorded with two cameras; (b) performance outcomes would be evaluated by the head coach of the tennis club; (c) performance results would be disclosed to all tennis club members; (d) if the participants performed significantly worse than others, they would be asked to participate in another experiment later; and (e) the reward money would be decreased by 300 yen per serve based on the results (down from a maximum of 3,000 yen). In (e), if the score was 5 or 6, the reward would be maintained. However, if the score were 4 or less, the reward would be reduced to 300 yen per serve (down from 3,000 yen). Therefore, the maximum reward would be 3,000 yen and the minimum 0 yen. The experimenter announced the score results and the amount earned aloud. All participants were debriefed after the experiment.

### 2.4 Measures

#### 2.4.1 Psychological anxiety

We gaged cognitive and somatic state anxiety using the Mental Readiness Form-3 (MRF-3; Krane, 1994). Each item was scored on an 11-point Likert scale ranging from 1 (*not worried*) to 11 (*worried*) for cognitive anxiety, and from 1 (*not tense*) to 11 (*tense*) for somatic anxiety.

#### 2.4.2 Serving scores

We quantified each serve outcome based on the area launched into the service box on the opposite side (Figure 1). We also counted the total number of achievements in the highest-ranked area (i.e., 6 points) that the participant served.

#### 2.4.3 Landing positions

The tennis court was video-recorded from a height of 3 m using a video camera (SONY FDR-AX60) at 120 frame-per-second (fps). We applied a direct 2D linear transformation (Abdel-Aziz et al., 1971) to the offline image data using Frame DIAS V (DKH, Japan) to obtain the ball landing positions. We calibrated the court with 70 points (10 on the x-axis and 7 on the y-axis) at 1-m intervals, and we defined the interaction between the center-service line and the net as the origin. We set the 2D coordinates as the x-axis for the centerline direction and the y-axis for the net direction. The error between the calibration and measured points was less than 0.01 m on both the x- and y-axes. After excluding net trials, we calculated the variable error (VE) for the x- and y-axes and the bivariate variable error (BVE) across tests to evaluate the participant’s variability in the landing positions (Hancock et al., 1995). We completed this using the following Equations (1–3), where the coordinates of each trial are *X_i_* and *Y_i_*, and the mean coordinates are *X_c_* and *Y_c_*:


(1) $$VEx=\;{\left[\frac{1}{n}\overset{n}{\underset{i=1}{\sum }}{\left(X_{i}-X_{c}\right)}^{2}\right]}^{1/2}$$

(2) $$VEy=\;{\left[\frac{1}{n}\overset{n}{\underset{i=1}{\sum }}{\left(Y_{i}-Y_{c}\right)}^{2}\right]}^{1/2}$$

(3) $$BVE=\;{\left[\frac{1}{n}\overset{n}{\underset{i=1}{\sum }}{\left(X_{i}-X_{c}\right)}^{2}+{\left(Y_{i}-Y_{c}\right)}^{2}\right]}^{1/2}$$

#### 2.4.4 Serving kinematics

We recorded the serving motion from the right sagittal plane 9 meters away using a high-speed camera (NAC Image Technology, MEMORECAM Q2m) at 250 fps. A marker was attached to the top of each racket. We performed the offline analysis using MOVIAS Neo ver. 3.0 (NAC Image Technology) by manually digitizing the markers and balls. We set the 2D coordinates as the x-axis for the net direction and the y-axis for the vertical direction. We calculated the ball speed, racket speed, and impact height. Based on the vertical and horizontal coordinates of the ball center after the impact, we defined the ball speed as the average of the resultant velocity in five frames after impact. Similarly, the racket speed was the average of the resultant velocities of the marker in the five frames before impact. We defined the impact height as the y-axis coordinate of the ball at impact when the floor was set to zero.

### 2.5 Statistical analysis

We subjected all measurements—including psychological states, serve performance (i.e., scores, the number of point 6 achievements, VE, and BVE), and kinematics (i.e., ball speed, racket speed, and impact height)—to two-way mixed-design analysis of variance (ANOVA) with a group factor (no-contraction/contraction) and a repeated measures factor of the test (pre−/pressure). When we found an interaction, we conducted multiple comparison tests by applying the Bonferroni correction. The effect size was expressed as partial eta squared (η_p_^2^). We performed statistical analyses using SPSS Statistics ver. 28 (IBM Corp. NY, Armonk, United States), with a significance level of 5%.

## 3 Results

### 3.1 Psychological scales

A two-way ANOVA confirmed an increase in somatic anxiety during the pressure test (*M* = 5.38, *SD* = 2.40) compared to the pre-test (*M* = 3.45, *SD* = 2.40) [*F* (1, 38) = 23.912, *p* < 0.001, η_p_^2^ = 0.442]. The interaction between group and test was also significant [*F*(1, 38) = 4.392, *p* = 0.043, η_p_^2^ = 0.442]. Simple main effect analyses demonstrated that somatic anxiety increased from the pre- to the pressure test and was significant for the contraction group (*p* < 0.001) and marginally significant for the no-contraction group (*p* = 0.055). Cognitive anxiety did not differ between the two tests [*F*(1, 38) = 2.533, *p* = 0.120, η_p_^2^ = 0.062] or groups [*F*(1, 38) = 1.561, *p* = 0.219, η_p_^2^ = 0.039].^1^

### 3.2 Performance

**Figure 4:**
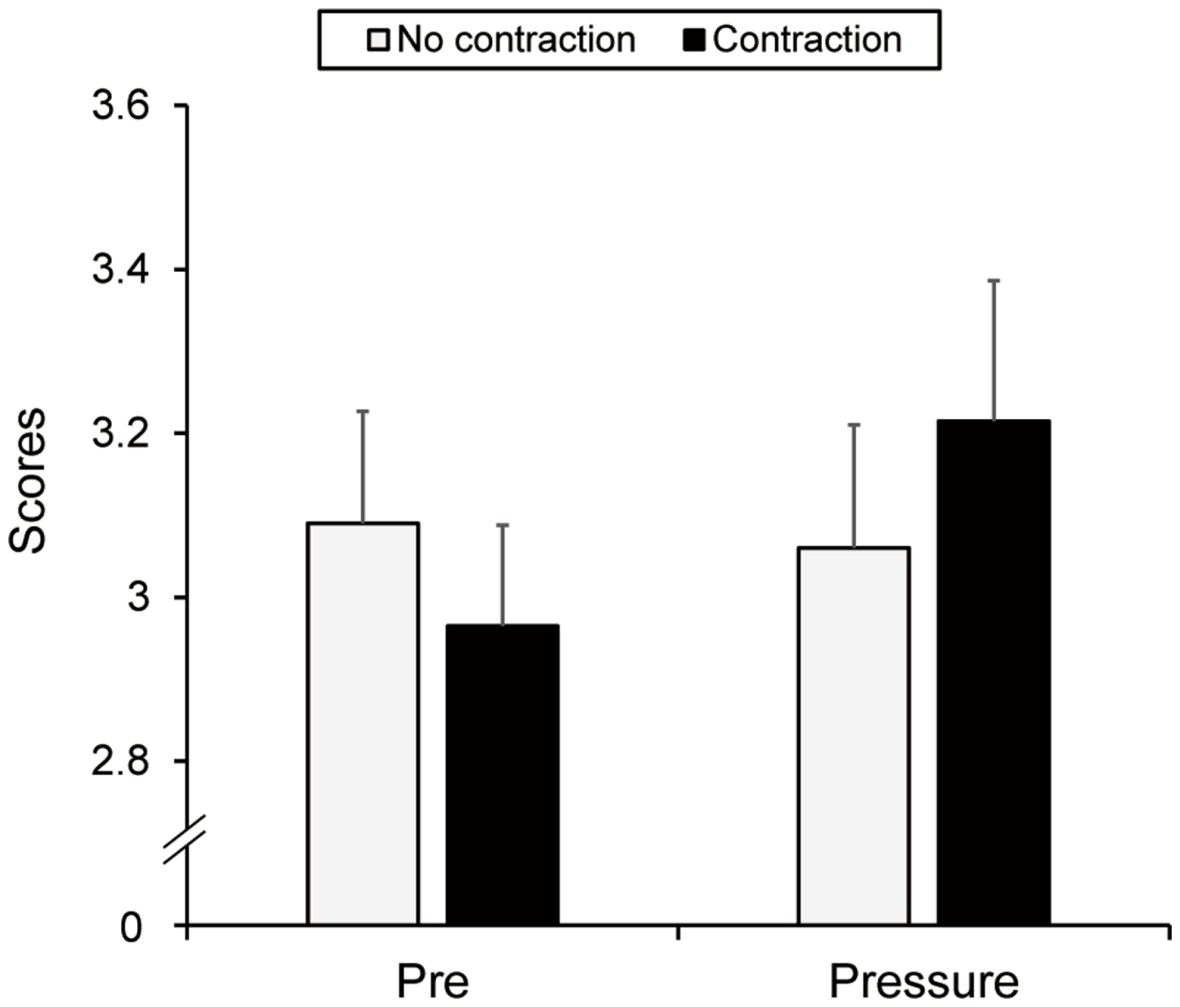
The scores of the tennis serve in the no-contraction and contraction groups on the pre- and pressure tests. The error bars indicate standard errors.

Figure 4 outlines the performance scores. A two-way ANOVA revealed no effects of group [*F*(1, 38) = 0.008, *p* = 0.929, η_p_^2^ = 0.000] or test [*F*(1, 38) = 0.720, *p* = 0.401, η_p_^2^ = 0.019]. The interaction between group and test was not significant [*F*(1, 38) = 1.167, *p* = 0.287, η_p_^2^ = 0.030].

**Figure 5:**
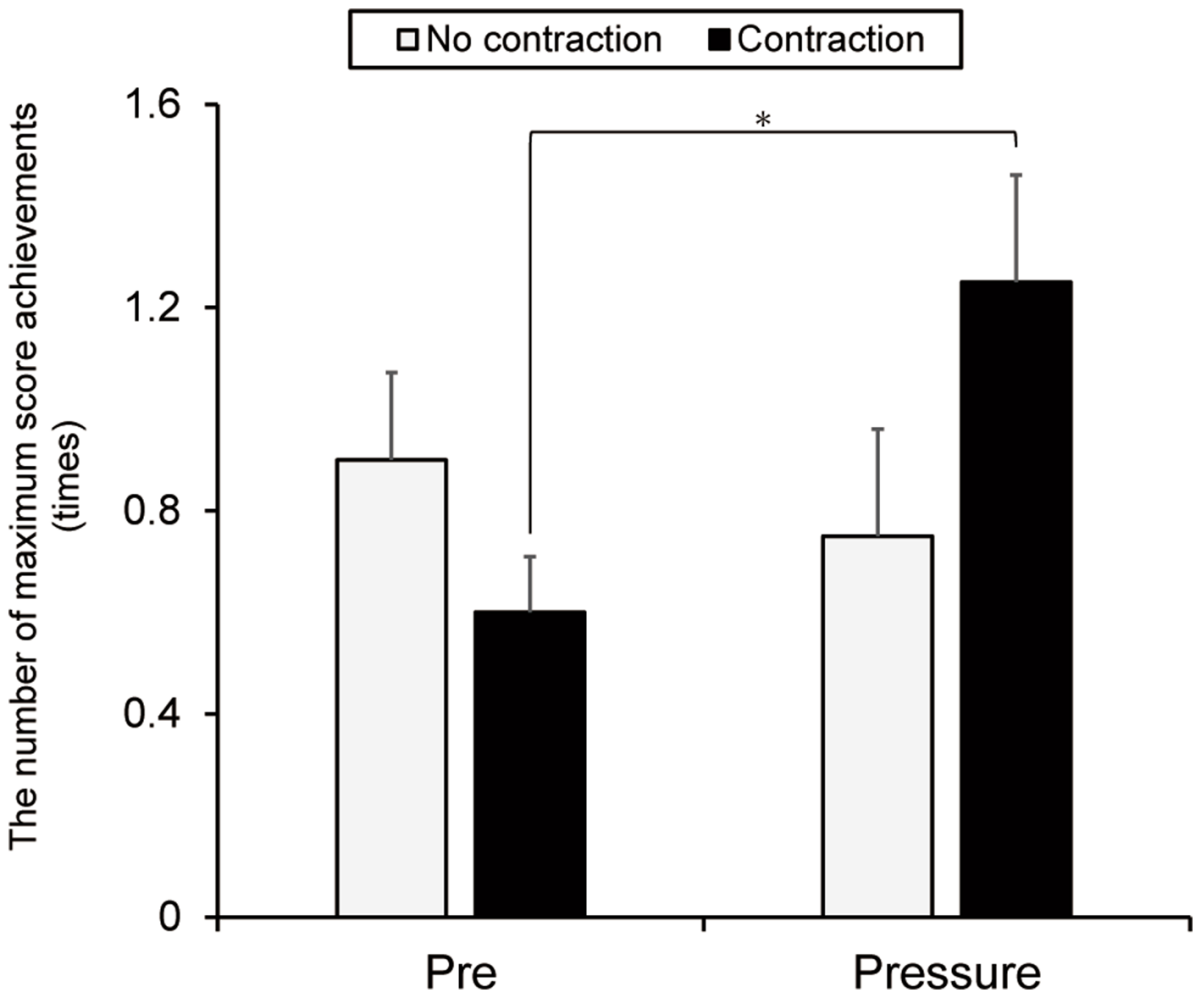
The number of maximum score achievements in the no-contraction and contraction groups on the pre- and pressure tests. The error bars indicate standard errors. *p < 0.05.

Figure 5 presents the number of maximum scores achieved. Neither a main effect of group [*F*(1, 38) = 0.310, *p* = 0.581, η_p_^2^ = 0.008] nor test [*F*(1, 38) = 1.724, *p* = 0.197, η_p_^2^ = 0.043] was found. However, the interaction between group and test was significant [*F*(1, 38) = 4.414, *p* = 0.042, η_p_^2^ = 0.104]. Simple main effect analyses demonstrated no group difference in the pre-test (*p* = 0.159). However, the contraction group improved the number of maximum score achievements in the pressure test (*M* = 1.25 times, *SD* = 0.94) compared to the pre-test (*M* = 0.6 times, *SD* = 0.49) (*p* = 0.021). The no-contraction group did not demonstrate any improvement in the number of maximum scores (pre-test, *M* = 0.9 times, *SD* = 0.76; pressure test, *M* = 0.75 times, *SD* = 0.94, *p* = 0.581).^2^

Table 1 presents the VE and BVE results. For VE (*x*), a two-way ANOVA revealed neither a main effect of group [*F*(1, 38) = 2.720, *p* = 0.107, η_p_^2^ = 0.067] nor test [*F*(1, 38) = 0.002, *p* = 0.968, η_p_^2^ = 0.000]. There was no interaction [*F*(1, 38) = 1.056, *p* = 0.311, η_p_^2^ = 0.027]. For VE (*y*), a two-way ANOVA indicated neither a main effect of group [*F*(1, 38) = 0.016, *p* = 0.901, η_p_^2^ = 0.000] nor test [*F*(1, 38) = 0.135, *p* = 0.715, η_p_^2^ = 0.004]. There was no interaction between group and test [*F*(1, 38) = 0.151, *p* = 0.700, η_p_^2^ = 0.004]. For BVE, neither an effect of group [*F*(1, 38) = 1.959, *p* = 0.170, η_p_^2^ = 0.049] nor test [*F*(1, 38) = 0.009, *p* = 0.923, η_p_^2^ = 0.000] was found. There was no interaction [*F*(1, 38) = 1.307, *p* = 0.260, η_p_^2^ = 0.033].

**Table 1:** Mean (*SD*) of the VE, BVE, and kinematics measures in the no-contraction and contraction groups on the pre- and pressure tests.

Measures (range)	Pre-test		Pressure test	
	No contraction	Contraction	No contraction	Contraction
VE x (m)	0.92 (0.31)	0.83 (0.25)	0.98 (0.45)	0.78 (0.24)
VE y (m)	0.60 (0.22)	0.62 (0.21)	0.60 (0.21)	0.59 (0.16)
BVE (m)	1.12 (0.31)	1.05 (0.27)	1.17 (0.44)	0.99 (0.24)
Ball speed (km/h)	112.9 (9.5)	111.2 (10.5)	116.3 (10.7)	115.2 (11.7)
Racket speed (km/h)	125.6 (11.6)	127.7 (17.7)	127.1 (11.5)	128.5 (18.1)
Impact height (m)	2.46 (0.14)	2.50 (0.15)	2.47 (0.13)	2.51 (0.15)

SD, standard deviation; VE, variable error; BVE, bivariate variable error; m, meter; km/h, kelometer per hour.

### 3.3 Kinematics

The kinematics data are summarized in Table 1. Both the ball and racket speeds were greater on the pressure test than on the pre-test [ball speed, *F*(1, 38) = 33.489, *p* < 0.001, η_p_^2^ = 0.468; racket speed, *F*(1, 38) = 6.763, *p* = 0.013, η_p_^2^ = 0.151]. These did not differ between the two groups [ball speed, *F*(1, 38) = 0.171, *p* = 0.681, η_p_^2^ = 0.004; racket speed, *F*(1, 38) = 0.137, *p* = 0.713, η_p_^2^ = 0.004]. No interactions were found [ball speed, *F*(1, 38) = 0.187, *p* = 0.668, η_p_^2^ = 0.005; racket speed, *F*(1, 38) = 0.537, *p* = 0.468, η_p_^2^ = 0.014].

For impact height, neither main effect of group [*F*(1, 38) = 0.550, *p* = 0.463, η_p_^2^ = 0.014] nor test [*F*(1, 38) = 2.079, *p* = 0.158, η_p_^2^ = 0.052] was found. There was no interaction [*F*(1, 38) = 0.003, *p* = 0.958, η_p_^2^ = 0.000].

## 4 Discussion

We aimed to investigate the effects of grasping materials with the left hand prior to a tennis second serve; a crucial skill players must execute during games. This material can be sewn into sportswear to facilitate easy access during a game. We found beneficial effects of the left-hand contraction (i.e., an increased number of maximum score achievements), extending a preliminary analysis that demonstrated null effects on performance indices (Masaki, 2022).

### 4.1 Serving scores

We hypothesized that the left-hand contraction could prevent choking under pressure based on previous findings in a series of experiments of Beckmann’s group (Beckmann et al., 2013; Gröpel and Beckmann, 2017; Beckmann et al., 2021). However, serve performance did not deteriorate with pressure manipulation, suggesting a failure of choking induction. Therefore, we failed to test the choking intervention, although participants perceived somatic anxiety during the pressure test.

Interestingly, the left-hand contraction group increased the number of maximum score achievements, supporting the assertion that the left-hand contraction may not only prevent choking but also enhance performance under pressure (Beckmann et al., 2013, Study 2; Gröpel and Beckmann, 2017, Study 2; Mesagno et al., 2019, Study 2). Our results reconfirmed the beneficial effects of the left-hand contraction on tennis serve performance, apart from the context of choking intervention. Beckmann et al. (2021) reported the effectiveness of left-hand contraction in preventing choking among skilled tennis players. Therefore, our data further support the evidence of the beneficial effects of left-hand contraction on enhancing tennis serve performance.

Performance enhancement under pressure is referred to as a “clutch” (Otten, 2009) that is conceptually opposite to the choking phenomenon. The occurrence of a clutch can be ascribed to a high level of athletes’ perceived control (Otten, 2009), which is an element of implicit knowledge. Based on Seger’s (1994) description, Otten (2009) defined perceived control as knowledge derived from the accurate prediction of subsequent stimuli or the ability to control the values of variables. The left-hand contraction may be associated with an improved sense of perceived control.

Given the occurrence of clutches, participants may have perceived increased somatic anxiety as optimal tension rather than a hindrance to their actions. An optimal level of anxiety may increase motivation properly (Eysenck and Calvo, 1992). Cheng et al. (2009) pointed out that success relies on whether individuals can interpret current pressure as a positive event. It is possible that the left-hand contraction also influences the interpretation of anxiety.

The occurrence of the clutch in our study may be due to the right dominant hemispheric asymmetry induced by the left-hand contraction (Harmon-Jones, 2006; Hirao and Masaki, 2019), which is known to be an appropriate brain state for expert athletes (e.g., Hatfield et al., 1984). Inhibition of the left hemisphere and prefrontal coactivation (i.e., weak T7-Fz connectivity, indicative of unconscious motor control) occurs after left-hand contractions (Hoskens et al., 2020). Furthermore, T7-Fz inhibition occurred more strongly for experts than for novices in golf (Gallicchio et al., 2016), and more for experts than for near experts in shooting (Deeny et al., 2003). Therefore, the inhibition of left hemispheric activity that manifests as the right-dominant hemispheric asymmetry is thought to be an ideal brain state for high performance.

### 4.2 Kinematic and two-dimensional indices

We posited that the left-hand contraction would conserve the serving kinematics even under pressure, whereas no contraction would disrupt the kinematics. However, this was not the case in this study. We observed no differences in movement kinematics between the two groups. Instead, we found improved performance accompanied by kinematic changes, although the direction of the changes was opposite to our prediction (i.e., the clutch). The kinematic analysis indicated that both groups exhibited faster ball and racket speeds during the pressure tests. In contrast to previous findings that have reported performance deterioration under pressure due to disrupted kinematics (e.g., decreased ball speed) (e.g., Sekiya and Tanaka, 2019), we noted better kinematic changes underlying the clutch phenomenon. Considering that the contraction group exhibited greater accuracy in serving toward the maximum scoring area, left-hand contraction may facilitate optimal serving attributes, such as increased ball speed and accuracy (e.g., Hernández-Davó et al., 2019), particularly under pressure.

The lack of differences in kinematics and serving variability (i.e., VE and BVE) between the groups can be attributed to analytical issues. The achievement of a successful tennis serve requires the coordination of several factors such as speed, impact angle, spin direction, and precision (Colomar et al., 2022). Mirifar et al. (2022) pointed out that the effect of unilateral hand contraction was fairly small and potentially insufficient to affect behavioral levels. Therefore, 3D kinematic analysis might be a good way to detect the beneficial effects of left-hand contraction (e.g., coordination among shoulder rotation, wrist and elbow flexion, or extension). Furthermore, in this study, the participants aimed at the target area instead of a specific target point, which was limited to the evaluation of several measures, including the mean radial error (MRE) and constant error (CE) from the target (Hancock et al., 1995). These indices are commonly used to assess tennis serve performance (Delgado-García et al., 2019; Hernández-Davó et al., 2019; Yamamoto et al., 2019; Beckmann et al., 2021). It would be fruitful to investigate the effects of hand contractions on these indices in future studies.

### 4.3 Practical implications

Our findings indicate that grasping a polyethylene material with the left hand would be helpful to enhance serving performance during critical, high-pressure matches in tennis. Previous studies have used soft balls (Beckmann et al., 2013; Gröpel and Beckmann, 2017; Mesagno et al., 2019) or tennis balls (Beckmann et al., 2021) for unilateral hand grasping. However, bringing these materials to real sports games is impractical, and grasping the ball *per se* likely interferes with motion. The developed wearable grasping material is likely to make the left-hand contraction more practical in real situations.

According to previous studies, left-hand contraction may be effective for expert or semi-expert players (Beckmann et al., 2013; Gröpel and Beckmann, 2017; Mesagno et al., 2019; Beckmann et al., 2021) but not for novices (Hoskens et al., 2020). Therefore, the effectiveness of our approach might be limited in skilled tennis players.

### 4.4 Limitations

Our study has several limitations. First, we could not assess the intervention effects during choking. Therefore, pressure manipulation must be reconsidered. Our participants were driven to achieve success through pressure manipulation. We manipulated pressure using a reward of 3,000 yen (approximately 20 USD). While this amount was set to increase the likelihood of participants seeking the reward, it may enhance the clutch phenomenon rather than induce choking. Second, we should point out the technical limitations of EEG recordings in real sporting situations. EEGs are vulnerable to muscular activities. Consequently, in our study, we did not know whether T7-Fz connectivity was reduced by the left-hand contraction. Future studies should employ a certain task (e.g., golf putting) during which EEGs can be recorded.

## 5 Conclusion

We discovered that grasping a polyethylene material with the left-hand enhanced serve performance, as evidenced by an increase in the number of maximum score achievements under pressure. This finding reinforces the notion that left-hand contraction may not only prevent choking but also contribute to the clutch phenomenon. Our study, utilizing embedded grasping material in sportswear rather than ball grasping, may expedite the practical application of left-hand contraction in real sporting scenarios.

## Data availability statement

The raw data supporting the conclusions of this article will be made available by the authors, without undue reservation.

## Ethics statement

This study was approved by The Waseda University Ethics Committee. The participants provided their written informed consent to participate in this study.

## Author contributions

KM: Writing – original draft. HM: Writing – review & editing.

## Footnotes

^1^We also conducted Wilcoxon signed-rank tests, which revealed a significant increase in somatic anxiety during the pressure test compared to the pre-test for both the contraction group (*Z* = 3.292, *p* < 0.001, *r* = 0.521) and the no-contraction group (*Z* = 2.042, *p* = 0.041, *r* = 0.322). However, cognitive anxiety did not differ between the tests for either the contraction group (*Z* = 1.207, *p* = 0.227, *r* = 0.036) or the no-contraction group (*Z* = 0.708, *p* = 0.479, *r* = 0.112).

^2^We also applied the Wilcoxon signed-rank test, which revealed that the number of maximum score achievements increased from the pre-test to the pressure-test in the contraction group (*Z* = 2.440, *p* = 0.015, *r* = 0.386). However, it did not change in the no-contraction group (*Z* = −0.441, *p* = 0.659, *r* = −0.07).
